# On the Causes and Treatment of Gangrene of the Leg

**Published:** 1906-05-12

**Authors:** Ernest W. Hey Groves

**Affiliations:** Assistant Surgeon to the Bristol General Hospital.


### Hospital Clinics.

### ON THE CAUSES AND TREATMENT OF GANGRENE OF THE LEG.

By Ernest W. Hey Groves, M.S., F.R.C.S., Assistant Surgeon to the Bristol General Hospital.

It is far easier to lay down general rules for a determination of the causes and treatment of gangrene than it is to apply these rules to any given case. Although the primary cause of the gangrene is generally evident, yet there are often many secondary factors which profoundly modify the course of the disease. It is usual, for instance, to speak of diabetes as a cause of gangrene, and yet it is hardly more than a predisposing agent, and when gangrene follows it is actually brought about by vascular occlusion, septic or other toxic agents acting directly on the tissues or by the intervention of a preliminary neuritis. And in many cases all these factors may be present in the same case. Thus diabetes causes neuritis, and an obliterative arteritis, these lead to anæsthesia, unnoticed injuries, and defective circulation. An injury in a part whose circulation is defective will lead to septic inflammation, thrombosis, and gangrene. And it is quite evident that the existence and extent of the vascular lesions, nerve lesions, and septic processes must each be taken into consideration before a rational treatment can be adopted. From a surgical point of view three questions have to be answered: Whether to amputate? When to amputate? Where to amputate?

I will relate three cases which have occurred recently in my practice which illustrate some of these points of difficulty.

1. A man aged 52, a butcher, had suffered from diabetes for five or six years. When I say "suffered" I mean that he had been told by a doctor that he had diabetes, but he had had only very transient general symptoms of the disease, together with a small perforating ulcer of his left foot. He had never taken the matter seriously, and after a short attempt at diet had declared that the cure was far worse than the disease and had taken no further precaution since. I saw him first in the spring of 1903, when he had a typical perforating ulcer under the ball of the right great toe. He had about 1 per cent. of sugar in the urine which rapidly disappeared under treatment. The ulcer was dressed and complete rest enjoined for a time. But he began to go about his business far sooner than I approved, and about a month later I was called to see him again and he had well-marked gangrene of the great toe extending to the prominence of the head of the metatarsal bone. At first the gangrene was of the moist variety, but as it was kept covered with antiseptic gauze it gradually shrivelled up and became dry. He had no pain whatever, and practically all his toes on the right foot were analgesic and partly anæsthetic. He had no knee-jerks. His pupils were normal, and there were no other signs of tabes. Within one week of the onset of gangrene, whilst I was deliberating what to do and when to do it, the whole of the foot swelled up and became quite dusky, and there was a perfectly distinct line of demarcation running across the dorsum of his foot, separating the dark skin from the normal. Nothing seemed more certain than that the gangrene was spreading, and Mr. Morton saw the patient with me. We both decided that amputation would be necessary, and it was only because we could not decide at the moment *where* to amputate that served to postpone the operation. His arteries seemed quite healthy and both dorsalis pedis and posterior tibial could be plainly felt pulsating. It seemed, therefore, reasonable to do a Symes amputation at the ankle-joint, but, on the other hand, this would have been so near the line of gangrene, and the generally accepted opinion is so definitely in favour of high amputation in diabetic gangrene, that we thought it would probably require an amputation below the knee. When the patient understood the point at issue he begged hard for an interval for consideration. Within three days the swelling of the foot had subsided, and within a fortnight the circulation in all the toes except the gangrenous great toe, had been restored, although the actual line which indicated "the high-water mark" of the threatened gangrene remained visible for months afterwards. I was able, therefore, to be content with a removal of the great toe only, at the metatarso-phalangeal joint. I performed this operation without the use of any anæsthetic either general or local, and he suffered no pain because of the analgesia. He made a good recovery and is now as active as ever. This shows the value of waiting in some cases for the real extent of gangrene to become definite before operating. The man's general condition was good and his blood-vessels sound. The cause of the gangrene was diabetes and neuritis and perforating ulcer. The degree of sepsis was reduced to a minimum, and therefore only a small amount of tissue was lost and an amputation performed actually at the line of gangrene led to the best results. It is well-known that gangrene spreads, but it is not sufficiently taught that it may and often does recede, or rather that the signs of gangrene recede. A part may present the five signs of gangrene—loss of pulsation, heat, sensation, function, and change of colour, and yet not be dead. If I had said that the foot presenting all these signs was dead and had removed it, I should have deprived him of a useful member which has served him well for the past five years.

2. Dr. F. asked me to see a lady of 49 who had a suppurating toe. She was very stout, and we thought had been addicted to alcoholic excess. She had been under Dr. F.'s care for eighteen months suffering from diabetes; but the symptoms had never been very pronounced and the sugar had lately quite disappeared from the urine. Ten days before I saw her she had hurt the second toe of her left foot and the three middle toes had swelled up and became inflamed. They had been opened in three different places and pus evacuated. On my first visit there was nothing in the appearance of the foot different from that of an ordinary thecal whitlow which had spread into the sole and dorsum and was discharging by several sinuses. She was so fat that the arteries could not be felt pulsating in either leg below the knee. There was no anæsthesia. It seemed quite clear that, owing either to an obliteration of the vessels or to the bad circulation associated with a fat heart, no attempt at healing was being made by the tissues or was likely to be made. And in spite of the fact that there was then no sugar in the urine, I advised removal of the foot at the ankle-joint. But the patient and her husband were so horrified by this advice that we consented to wait for three days, and in the meantime, under ether, we removed the three affected toes and opened up the sloughing tissues of the sole and dorsum of the foot. Three days later I received an urgent message to come prepared to amputate; but on my arrival the patient was deeply comatose, her urine was loaded with sugar, and the foot presented the signs of moist gangrene well above the ankle. We transfused her with three pints of saline fluid, which slightly revived her, but she died within forty hours. In this case, diabetes, a bad circulation, and sepsis all contributed to cause the gangrene, and I think she might have done better if, instead of the temporising operation, I had left her alone until she was willing to have the foot off at the ankle or even the knee. Because, whilst a very feeble circulation is sufficient for the healing of an aseptic wound—as my third case will show—a vigorous circulation is necessary for the repair of a large septic area. The causation of the coma in this case is a matter of great interest and importance. There were four factors which contributed to its production in an already diabetic patient. The mental shock of being told she must lose her leg, the physical shock of the operation, the chemical effect of the ether used as an anæsthetic, and the toxic effect of the septic products in the wounds.

Within recent years it has been proved that a condition very similar to that of diabetic coma may be produced by various acid toxins—the so-called acid-intoxication or acidæmia. And in such cases, which occur most often in children, a fatal result generally follows the administration of an anæsthetic. In some instances of operations performed for other diseases, *e.g.* appendicitis, this acid intoxication is produced apparently by the anæsthetic. It may result from the solvent effect of the ether or chloroform upon the fats, producing fatty acid products. If this theory be true then it would be better to use local anæsthesia for operations in cases of diabetic gangrene wherever this is possible.

3. A professional man of 40, had had two attacks of rheumatic fever within the past ten years. This had left him with well-marked signs of valvular disease of the heart, the aortic valve being chiefly affected. Since the last rheumatic attack, five years ago, he had suffered from a serious condition of heart-failure three times. The signs of this failure had been great prostration and tendency to faint, together with a very slow and irregular pulse. He had never had dropsy. On December 23, 1905, when he was in his best health, he was about to step into a cab when he was seized with such violent pain in his left leg that he fell and had to be carried into the house. The whole of the leg became cold and numb, and no pulse could be felt in it. The pain continued to be so severe as to require morphia. The toes all became dark and shrivelled and a definite limit to the gangrene crossed the dorsum of the foot about 1½ inches above the web of the toes. On December 30, a week after the onset of symptoms, the pain, which had been localised in the foot, suddenly spread up the leg. By January 1, 1906, the whole of the leg below the knee was very much swollen and exquisitely tender. At this time five distinct zones could be distinguished in the leg. The thigh was pulseless but otherwise normal. The knee and six inches below were swollen, very tender, but of natural colour. The upper part of the calf presented large purple and red patches of ecchymosis, the lower part of the calf, the ankle, and part of the foot were uniformly swollen, livid, and covered with blebs—evidently a condition of moist gangrene. The toes were shrivelled and contracted showing the tendons through the transparent skin. His temperature varied between 99° and 100° F., and his pulse was rather irregular, 60-80. The pain was so great and incessant that he could get no sleep without morphia, and he begged to have the leg off. I amputated the limb on January 3, removing it just above the femoral condyles. No tourniquet was applied, and not a single vessel bled a drop—in fact, as far as hæmorrhage was concerned, it might have been a post-mortem operation. I therefore removed a further part of the limb, cutting the femur at a junction of the middle and lower ⅓. The femoral artery and vein were firmly thrombosed, but the small vessels in the skin oozed although none spurted. The skin flaps were sewn up without a drainage tube. It seemed rather a hopeless proceeding to amputate through a region whose vessels were all thrombosed, but the alternative of removing the limb at the hip-joint was too severe for a patient with advanced heart disease. I expected the flaps would slough or that healing would be slow and tedious; but as a matter of fact no stump ever behaved in a more ideal way. Firm healing took place by first intention, the patient was allowed up three weeks after the operation. But even a month later no pulsation could be felt in any part of the left thigh, not even in the external iliac artery. In the situation of the latter, however, was a firm, rather tender cord.

No better illustration could be furnished than this case of how complex a matter gangrene of the leg may be. In the first place there must have been for a long time a bad circulation owing to the valvular lesion in the aorta. Then an embolus must have been detached from the aorta or from one of the dilated recesses of the left ventricle. This became lodged in the external iliac artery and was the primary cause of all the subsequent events. The first objective sign produced by the embolus was dry gangrene, and, as is usually the case, this only affected the terminal part of the extremity and not, as might have been anticipated, the whole limb. The leg must have been nourished merely by those small arteries by which the branches of the internal iliac anastomose with branches of the femoral, namely by the sciatic, gluteal, internal pudic, and obturator. Through the narrow anastomotic channels nothing but a slow, pulseless stream of blood can be driven which finds its way into the femoral artery below the embolus. This is enough for the greater part of the limb, but for the most distant parts, the toes with their small arteries and network of capillaries, the stream has not enough force, and in these parts no circulation takes place and dry gangrene results. The gangrene is dry because the veins still take blood from the parts while the arteries bring no more to them. A week later moist gangrene of the leg occurred. This was due to thrombosis in the veins of the leg running up to the popliteal vein, which was found firmly plugged at the time of operation. The thrombosis is caused by the very slow blood-stream returned to the veins. It causes gangrene by adding an insuperable block to this feeble arterial stream. The gangrene is moist because the blood is all imprisoned in the tissues. Moist and dry gangrene are very differently related to septic processes. Tissues drained of blood afford no opportunity for the development of bacterial activity and therefore septic inflammation takes no part in the development of the process. But tissues gorged with stagnant clotted blood form a perfect culture medium for pyogenic organisms which readily obtained access to them through the skin or circulation. So that dry gangrene may be described as death plus mummification, and moist gangrene as death plus putrefaction. And dry gangrene will remain stationary and the dead part be eaten into and removed by the active granulations of the living parts. If it spreads it will be by leaps and bounds, indicating the blocking of a fresh area of arteries. Moist gangrene, on the other hand, soon becomes a focus of septic activity which will invade the living tissues in its neighbourhood and by setting up fresh thrombosis, the process will gradually spread, and when it comes to an end it will be because an area of ulceration has been formed at the expense of the living tissue which has been destroyed by the activity of the bacteria in the dead part. Of course, in symptomatic, as distinguished from infective, gangrene, sepsis is not an original factor in the process either in the dry or moist variety. Therefore the first essential of treatment consists in rendering the parts as aseptic as possible, and the second in keeping them so. But when the difficulty of carrying this out in a large area of very painful skin is considered, it will be readily understood how soon large areas of moist gangrene must become infected in spite of all precautions.

As to the treatment of these two conditions when due to simple vascular lesions. If dry gangrene be due to occlusion of a main vessel at a distance from the dead tissue, as in the third case, we must first wait for a line of ulceration to form, and then amputate immediately above that line. If amputation be performed before the line of ulceration is present, these parts will be removed which might recover, as was illustrated by the first case I mentioned. The actual vessels of the part are healthy, therefore there is no need to remove more than the dead tissue. If, on the other hand, dry gangrene be due to thrombosis of diseased and obliterated arteries as in senile gangrene, and amputation be performed, it will have to be through an area where the vessels are more healthy and can respond to the dilatation demanded by the healing of flaps. In moist gangrene the amputation must in all cases be well above the involved area and away from the septic district. It is much more imperative also to act without undue delay, because, as I have explained above, the process generally spreads and involves more and more of the living tissues. How should the line of amputation be related to the embolus in a case of gangrene due to the latter? The answer to this question (illustrated in a very perfect manner by my third case) is that the position of the embolus should be ignored and amputation be performed in relation to the presence and extent of the dry or moist gangrene on the principles already stated. It is not the condition of the main vessel but that of the small vessels upon which the vitality of the parts depend. And if there is sufficient blood circulating in the limb at the point of amputation to keep it alive there is enough blood to bring about repair. Of course, this may lead in some cases to a sloughing of flaps because the damage done to the tissues by the amputation may cause a degree of inflammation which leads to an arrest of the circulation. But it is certainly right to give the living tissues the chance of healing, and by so doing to save a useful limb.

Of all the factors associated with gangrene, and of all the elements which must lead to a decision as regards amputation, two are of pre-eminent importance. These are the state of the small blood-vessels and the presence of sepsis. So that provided the small vessels are healthy and the parts aseptic, amputation may be performed almost anywhere through living tissues with a good prospect of success, even though the blood-stream is slow and pulseless.

I have only related three cases, but I think they suffice to show how very complex are the conditions underlying the production of gangrene and how each case must be carefully considered before a correct estimate of treatment can be arrived at.

